# Incidence of viral hepatitis in Brazil from 2009 to 2018: an epidemiological study of confirmed cases of viral hepatitis

**DOI:** 10.1590/0037-8682-0089-2020

**Published:** 2020-12-21

**Authors:** Alessandra Pereira da Silva, Andresa Pereira Silva, Lohana Marques Leal de Souza, Natália de Oliveira Peixoto, Evonnildo Costa Gonçalves, João Lídio da Silva Gonçalves Vianez

**Affiliations:** 1 Instituto Evandro Chagas, Centro de Inovações Tecnológicas, Programa de Pós-Graduação Stricto Sensu em Virologia, Ananindeua, PA, Brasil.; 2 Universidade Federal do Pará, Instituto de Ciências da Saúde, Especialização em Hematologia e Imunologia, Belém, PA, Brasil.; 3 Universidade Federal do Pará, Faculdade de Formação e Desenvolvimento do Campo, Abaetetuba, PA, Brasil.; 4 Universidade Federal do Pará, Instituto de Ciências Biológicas, Laboratório de Tecnologia e Biomolecular, Belém, PA, Brasil.

**Keywords:** Human Viral Hepatitis, Epidemiology, Public health, Hepatitis A, Hepatitis B, Hepatitis C

## Abstract

**INTRODUCTION:**

Viral hepatitis is a major public health problem. It is necessary to understand the epidemic, verifying the combination of biological and demographic characteristics.

**METHODS:**

This is an analytical ecological and epidemiological study. Confirmed case data from the Notification Disease Information System (SINAN) were used.

**RESULTS:**

From 2009-2018, SINAN confirmed 404,003 viral hepatitis cases in Brazil, with 12.49%, 37.06%, and 48.28% cases of hepatitis A, B, and C, respectively.

**CONCLUSIONS:**

In Brazil, 4,296 deaths were associated with viral hepatitis, of which 36.66% were associated with acute hepatitis B. The proportional distribution of cases varied among the five Brazilian regions.

Viral hepatitis is an inflammation of the liver caused by a virus. According to the World Health Organization (WHO), human viral hepatitis is classified based on the following etiological agents: *Hepatovirus A* (HAV), *Hepatitis B virus* (HBV), *Hepacivirus C* (HCV), *Hepatitis delta virus*, and *Orthohepevirus A* ^1^ ^-^ ^3^.

Viral hepatitis is a major public health concern. At least 400 million people are chronically infected with HBV and HCV worldwide, and 1.4 million people are infected annually with HAV^4^. Chronic viral hepatitis, initially silent, takes several years to develop complications. It is believed that 57% of cases of liver cirrhosis and 78% of cases of liver cancer are directly related to HBV and HCV infections^5^. In highly endemic regions, over 90% of children are infected with HAV at 10 years of age, although few develop complications^6^. Finally, it is estimated that 1.5 million deaths are related to viral hepatitis.

Therefore, it is necessary to understand viral hepatitis epidemics and their particularities by verifying the association between biological and socioeconomic risk factors and epidemiological characteristics. For investigating the incidence of confirmed cases of viral hepatitis in the five Brazilian regions that use the public health system, we used the data generated by the Disease and Notification Information System (SINAN).

This is an analytical ecological and epidemiological study, the objective of which was to assess socio-demographic variables such as education, race, gender, age group, and the source of infection. For the present study, we used the data of confirmed cases available from the Department of Informatics of the Unified Health System, based on notified regions from SINAN, obtained via a generic public domain tabulator called TABNET v. 4.14., during 2009 to 2018. The tool used to generate graphics was RStudio Version 1.2.5019^7^. 

In Brazil, from 2009 to 2018, out of 409,003 confirmed cases of hepatitis, 12.49% of cases were infections of HAV (Figure 1A). The main routes of infection with HAV are the fecal-oral route, inter-human contact, and transmission through contaminated food and water. The environmental stability of HAV and the high amount of virus present in the feces of infected individuals also influence dissemination viral. Parenteral transmission is uncommon but may occur if the donor is in the viremia phase of the incubation period. Dissemination is related to the reduction of basic sanitation infrastructure and hygiene conditions^8^.

**FIGURE 1:**
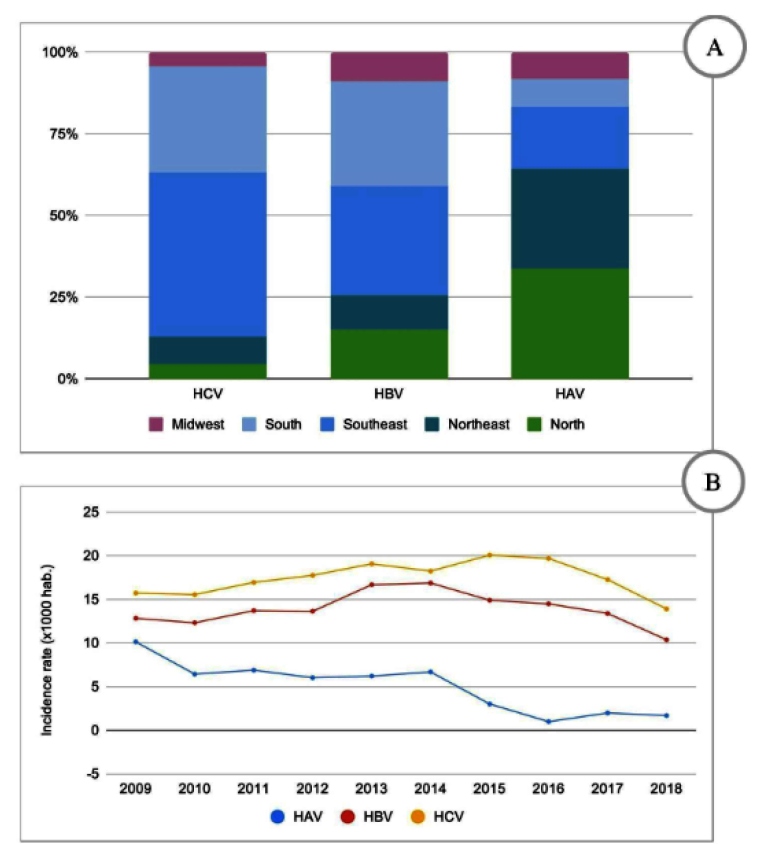
Cases of viral hepatitis. **A:** proportional distribution of cases varies among the five Brazilian regions; **B:** viral hepatitis incidence rates.

Until 2018, the incidence of hepatitis A remained higher in children under 10 years of age compared to other age groups, regardless of gender. Regarding the cumulative cases of hepatitis A in the country, those that occurred in this age group accounted for 30.6% (2009 to 2018, view Supplementary data in Webappendix Table 1). This is in agreement with the Brazilian Ministry of Health, which reported in 2018 that in the regions with more sanitation infrastructure and water treatment problems, people are exposed to HAV at earlier ages and present subclinical or anicteric forms of the disease^8^ ^,^ ^9^. In many cases, hepatitis A is self-limiting and benign, with severe acute liver failure occurring in less than 1% of cases^9^.

In this study, most cases of hepatitis A were concentrated in the north and northeast regions of the country, representing 33.6% and 30.8% of all confirmed cases in the period from 2009-2018, respectively, as shown in Figure 1B. These results are in agreement with Brazilian Institute of Geography and Statistics Foundation data related to access to basic sanitation services, which indicate that this is still an issue that poses a challenge in northern and northeastern Brazil, according to data from the National Continuous Household Sample Survey Characteristics of Residents and Households. The two regions remain below the national average for water supply, sewage, and garbage collection^9^.

The Epidemiological Bulletin of Viral Hepatitis by the Brazilian Ministry of Health, published in 2018, provided a general overview of confirmed cases of hepatitis B from 1999 to 2017, and the results obtained confirm the findings of the present study. This shows that most confirmed cases of HBV were in the southeast region, followed by the south, north, northeast, and midwest (Supplementary data in Webappendix Table 2)^8^.

In addition, on stratification of the data from 2007 to 2017, a higher prevalence of cases was found among males (54.4%) and those aged between 25 and 39 years (36.8%). In 2017, there was a higher prevalence of confirmed cases in self-identifying white people (46.5%), followed by brown, black, yellow, and indigenous, at 41.2%, 10.1%, 1.5%, and 0.7%, respectively^8^. These data, once again, corroborate the findings of this study, in which the majority of infected individuals are men, aged between 20 and 39 years, self-identifying as white.

The Pan American Health Organization (PAHO) reported that while the number of hepatitis-related deaths is increasing globally, new HBV infections are declining, and WHO links this reduction in the occurrence to increased vaccination coverage against this virus in children^10^. Additionally, in this study, it is possible to relate the age group with the highest occurrence of hepatitis B cases found in this study (20 to 39 years) with what PAHO understands as pre-vaccine, which covers the period from the 1980s to the beginning of 2000. People born in this period are more likely to be infected with the virus^11^.

Bandeira et al. (2018), reported that hepatitis B was the second most reported hepatitis in the state of Minas Gerais from 2010 to 2017, with 39% of the total cases; among these, the main route of transmission was the sexual route, followed by transfusion. The authors noted the importance of hepatitis caused by HBV and HCV based on the large number of infected individuals and the potential chronicity of the pathologies caused by these viruses. The state of Minas Gerais belongs to the southeastern region of Brazil, which, in this study, had the highest percentage of hepatitis B cases, and among the reported cases, the vast majority of patients had chronic hepatitis (81.8%)^12^.

PAHO ratifies the importance of prevention, early diagnosis, treatment, and attention to hepatitis as a way of preventing the chronicity of the disease with respect to its evolutions such as cirrhosis and liver cancer, which in 2018, were considered the fourth leading cause of male mortality and the seventh leading cause of female cancer mortality in the Americas, which is a notable public health problem^13^.

A study conducted in the city of Minas Gerais showed the main causes of HBV contamination^14^. In this study, the authors noted that among those infected, 19.8% reported having three or more sexual partners, 10.7% reported having sexual contact with HBV carriers, and 5.4% reported having occupational contact with HBV patients. These data demonstrate the relevance of educational campaigns about hepatitis B as well as the awakening from intellectual inertia of the population about the disease.

From 2009 to 2018, 195,039 cases of hepatitis C with one of the anti-HCV or HCV-RNA reactive markers were reported in Brazil. In the analysis of the distribution of cases with anti-HCV and HCV-RNA reagents by region, 50.65%, 32.18%, 8.19%, 4.59%, and 4.39% of these occurred in the southeast, south, northeast, north, and midwest regions, respectively (Figure 1). In 2018, the state capitals with the largest confirmed cases of hepatitis C were São Paulo, Rio Grande do Sul, and Rio de Janeiro, with 35%, 20.6%, and 8.8% of cases, respectively. Considering only the confirmed cases of hepatitis C, 57% occurred in males and 43% in females. Throughout the study period, it was observed that most cases of hepatitis C occurred in individuals above 40 years of age, and this trend was observed in both genders (Supplementary data in Webappendix Table 3).

In the last decade, hepatitis C has been the leading cause of death in Brazil among the types of viral hepatitis, mainly affecting adults over 40 years. The Epidemiological Bulletin of Viral Hepatitis, published by the Brazilian Ministry of Health in 2012, reported that between 2000 and 2011, 30,931 deaths from hepatitis C, with 16,896 as the underlying cause and 14,035 as an associated cause, were reported in the Mortality Information System, most of them occurring in the southeast (57.5%) and south (25.5%) regions^8^.

HCV infection is basically transmitted through blood. Most carriers became infected via transfusions performed before 1992 (when there were no specific tests for virus detection in blood banks) or by sharing needles and syringes, especially among injecting drug users. In the United States, over the past 5 years, 60% of the 25 to 40,000 people who have become infected with HCV have acquired it through injecting drug use^15^. In this study, we observed that 55.7% of the individuals with reported cases were unaware of the origin of the infection (transmission during pregnancy, unprotected sex, or use of contaminated blood in domestic or hospital materials are some possibilities). Because notifications are not complete, it is impossible to detail the main risk factors in our population^16^.
