# Supplementary material for: Incidence of viral hepatitis in Brazil from 2009 to 2018: an epidemiological study of confirmed cases of viral hepatitis
Source: Rev Soc Bras Med Trop. 2020 Dec 21;54:e00892020. doi: 10.1590/0037-8682-0089-2020 (PMC7747823; doi:10.1590/0037-8682-0089-2020)
Supplement: Supplementary file 1 [file 1678-9849-rsbmt-54-e00892020-suppl1.pdf]

Webappendix Table 1. Descriptive profile of the population with HAV.

	2009 n=10118		2010 n=6425		2011 n=6878		2012 n=6011		2013 n=6196		2014 n=6667		2015 n=3001		2016 n=974		2017 n=1972		2018 n=1678	
	n	%	n	%	n	%	n	%	n	%	n	%	n	%	n	%	n	%	n	%
**Region**																				
North	2413	23.8	1860	28.9	2632	38.3	2535	42.2	2178	35.2	2966	44.5	1498	49.9	382	39.2	191	9.7	145	8.6
Northeast	3780	37.4	2194	34.1	2111	30.7	1569	26.1	2456	39.6	2123	31.8	632	21.1	218	22.4	159	8.1	115	6.9
Southeast	1419	14.0	895	13.9	1121	16.3	1091	18.2	976	15.8	813	12.2	475	15.8	193	19.8	1379	69.9	1138	67.8
South	1190	11.8	1002	15.6	477	6.9	388	6.5	248	4.0	188	2.8	154	5.1	110	11.3	186	9.4	222	13.2
Midwest	1316	13.0	474	7.4	537	7.8	428	7.1	338	5.5	577	8.7	242	8.1	71	7.3	57	2.9	58	3.5
**Sex**																				
man	5366	53.0	3454	53.8	3683	53.5	3185	53.0	3316	53.5	3564	53.5	1678	55.9	526	54.0	1464	74.2	1157	69.0
woman	4750	46.9	2970	46.2	3195	46.5	2825	47.0	2879	46.5	3101	46.5	1323	44.1	448	46.0	508	25.8	521	31.0
unknown	2	0.02	1	0.0	0	0	1	0.0	1	0.0	2	0.0	0	0.0	0	0.0	0	0.0	0	0
**Age group (years)**																				
<10 anos	5488	54.2	3223	50.2	3352	48.7	2929	48.7	2868	46.3	3141	47.1	1163	38.8	247	25.4	115	5.8	99	5.9
10-19 anos	2903	28.7	1886	29.4	2074	30.2	1819	30.3	1864	30.1	2107	31.6	905	30.2	216	22.2	222	11.3	237	14.1
20-39 anos	1269	12.5	930	14.5	1039	15.1	905	15.1	1048	16.9	989	14.8	581	19.4	262	26.9	1183	60.0	949	56.6
40 ou mais	454	4.5	383	6.0	411	6.0	356	5.9	414	6.7	429	6.4	352	11.7	249	25.6	451	22.9	393	23.4
unknown	4	0.04	3	0.0	2	0.0	2	0.0	2	0.0	1	0.0	-	-	-	-	1	0.1	-	-
**Race**																				
white	2894	28.6	1721	26.8	1597	23.2	1232	20.5	1200	19.4	1135	17.0	594	19.8	229	23.5	860	43.6	713	42.5
black	446	4.4	335	19.5	358	5.2	280	4.7	275	4.4	305	4.6	117	3.9	50	5.1	86	4.4	107	6.4
yellow	83	0.8	49	0.8	40	0.6	45	0.7	41	0.7	55	0.8	24	0.8	8	0.8	17	0.9	14	0.8
mulato	5186	51.3	3429	53.4	4019	58.4	3576	59.5	3547	57.2	4346	65.2	1993	66.4	559	57.4	551	27.9	530	31.6
indigenous	103	1.0	97	1.5	73	1.1	105	1.7	214	3.5	178	2.7	49	1.6	18	1.8	7	0.4	5	0.3
unknown	1406	13.9	794	12.4	791	11.5	773	12.9	919	14.8	648	9.7	224	7.5	110	11.3	451	22.9	309	18.4
**Education (years)**																				
illiterate	53	0.5	47	0.7	45	0.7	42	0.7	58	0.9	60	-	43	1.4	17	1.7	21	1.1	9	0.5
1 to 4	2375	23.5	1376	21.4	1395	20.3	1130	18.8	1171	18.9	1409	21.1	577	19.2	153	15.7	139	7.0	146	8.7
5 to 8	1437	14.2	913	14.2	953	13.9	904	15.0	793	12.8	993	14.9	503	16.8	165	16.9	182	9.2	175	10.4
9 to 11	661	6.5	491	7.6	571	8.3	504	8.4	487	7.9	641	9.6	350	11.7	170	17.5	332	16.8	297	17.7
≥12	149	1.5	96	1.5	164	2.4	130	2.2	117	1.9	150	2.2	111	3.7	43	4.4	389	19.7	283	16.9
unknown	5443	53.8	3502	54.5	3750	54.5	3301	54.9	3570	57.6	3414	51.2	1417	47.2	426	43.7	909	46.1	768	45.8
**Source of infection**																				
sexual	71	0.7	34	0.5	50	0.7	54	0.9	50	0.6	49	0.7	48	1.6	24	2.5	181	9.2	98	5.8
transfusion	9	0.1	7	0.1	3	0.0	9	0.1	4	0.1	3	0.0	3	0.1	5	0.5	5	0.3	2	0.1
injection drug use	8	0.1	5	0.1	3	0.0	6	0.1	7	0.1	-	-	2	0.1	-	-	9	0.5	7	0.4
vertical transmission	1	0.0	2	0.0	4	0.1	2	0.0	2	0.0	2	0.0	1	0.0	-	-	-	-	1	0.1
work accident	1	0.0	2	0.0	1	0.0	1	0.0	2	0.0	1	0.0	4	0.1	-	-	-	-	1	0.1
hemodialysis	1	0.0	5	0.1	1	0.0	-	-	1	0.0	-	-	-	-	1	0.1	-	-	-	-
home	740	7.3	381	5.9	418	6.1	241	4.0	229	3.7	266	4.0	78	2.6	50	5.1	46	2.3	28	1.7

surgical treatment	6	0.1	3	0.0	3	0.0	2	0.0	1	0.0	2	0.0	1	0.0	2	0.2	5	0.3	1	0.1
dental treatment	109	1.1	50	0.8	50	0.7	41	0.7	23	0.4	25	0.4	20	0.7	9	0.9	13	0.7	14	0.8
person to person	252	2.5	159	2.5	273	4.0	224	3.7	201	3.2	170	2.5	42	1.4	7	0.7	54	2.7	36	2.1
oral/fecal	5597	55.3	3558	55.4	3895	56.6	3949	65.7	4301	69.4	4683	70.2	2237	74.5	573	58.8	656	33.3	713	42.5
others	240	2.4	149	2.3	177	2.6	58	1.0	38	0.6	31	0.5	24	0.8	28	2.9	32	1.6	25	1.5
unknown	3083	30.5	2070	32.2	2000	29.1	1424	23.7	1337	21.6	1435	21.5	541	18.0	275	28.2	971	49.2	752	44.8
**Federative unit**																				
Rondônia	84	0.8	77	1.2	105	1.5	21	0.3	57	0.9	127	1.9	63	2.1	20	2.1	5	0.3	25	1.5
Acre	71	0.7	100	1.6	402	5.8	619	10.3	302	4.9	223	3.3	97	3.2	72	7.4	37	1.9	14	0.8
Amazonas	904	8.9	693	10.8	729	10.6	391	6.5	675	10.9	1031	15.5	341	11.4	66	6.8	48	2.4	13	0.8
Roraima	243	2.4	104	1.6	67	1.0	123	2.0	129	2.1	129	1.9	42	1.4	18	1.8	9	0.5	22	1.3
Pará	470	4.6	364	5.7	672	9.8	732	12.2	702	11.3	770	11.5	458	15.3	143	14.7	47	2.4	51	3.0
Amapá	173	1.7	262	4.1	340	4.9	219	3.6	96	1.5	412	6.2	214	7.1	40	4.1	35	1.8	14	0.8
Tocantins	468	4.6	260	4.0	317	4.6	430	7.2	217	3.5	274	4.1	283	9.4	23	2.4	10	0.5	6	0.4
Maranhão	463	4.6	337	5.2	446	6.5	300	5.0	268	4.3	281	4.2	158	5.3	43	4.4	28	1.4	14	0.8
Piuaí	298	2.9	139	2.2	171	2.5	152	2.5	85	1.4	91	1.4	31	1.0	20	2.1	9	0.5	12	0.7
Ceará	407	4.0	243	3.8	134	1.9	196	3.3	224	3.6	117	1.8	48	1.6	15	1.5	18	0.9	19	1.1
Rio Grande do Norte	308	3.0	170	2.6	152	2.2	144	2.4	255	4.1	54	0.8	9	0.3	8	0.8	10	0.5	15	0.9
Paraíba	455	4.5	304	4.7	188	2.7	144	2.4	491	7.9	302	4.5	59	2.0	7	0.7	15	0.8	8	0.5
Pernambuco	682	6.7	328	5.1	342	5.0	268	4.5	520	8.4	570	8.5	74	2.5	37	3.8	17	0.9	14	0.8
Alagoas	277	2.7	231	3.6	319	4.6	145	2.4	229	3.7	157	2.4	115	3.8	37	3.8	18	0.9	4	0.2
Sergipe	112	1.1	61	0.9	89	1.3	56	0.9	42	0.7	72	1.1	15	0.5	6	0.6	7	0.4	2	0.1
Bahia	778	7.7	381	5.9	270	3.9	164	2.7	342	5.5	479	7.2	123	4.1	45	4.6	37	1.9	27	1.6

Minas Gerais	755	7.5	299	4.7	432	6.3	278	4.6	153	2.5	133	2.0	131	4.4	78	8.0	109	5.5	104	6.2
Espírito Santo	73	0.7	11	0.2	14	0.2	14	0.2	42	0.7	14	0.2	5	0.2	8	0.8	5	0.3	6	0.4
Rio de Janeiro	419	4.1	477	7.4	541	7.9	680	11.3	643	10.4	420	6.3	170	5.7	22	2.3	205	10.4	433	25.8
São Paulo	172	1.7	108	1.7	134	1.9	119	2.0	138	2.2	246	3.7	169	5.6	85	8.7	1060	53.8	595	35.5
Paraná	510	5.0	123	1.9	86	1.3	87	1.4	88	1.4	39	0.6	84	2.8	42	4.3	76	3.9	41	2.4
Santa Catarina	143	1.4	44	0.7	32	0.5	39	0.6	29	0.5	48	0.7	30	1.0	16	1.6	48	2.4	40	2.4
Rio Grande do Sul	537	5.3	835	13.0	359	5.2	262	4.4	131	2.1	101	1.5	40	1.3	52	5.3	62	3.1	141	8.4
Mato Grosso do Sul	267	2.6	41	0.6	51	0.7	46	0.8	27	0.4	115	1.7	32	1.1	8	0.8	8	0.4	6	0.4
Mato Grosso	309	3.1	129	2.0	220	3.2	86	1.4	142	2.3	327	4.9	149	5.0	37	3.8	16	0.8	33	2.0
Goiás	371	3.7	169	2.6	101	1.5	82	1.4	62	1.0	45	0.7	20	0.7	11	1.1	21	1.1	12	0.7
Distrito Federal	369	3.6	135	2.1	165	2.4	214	3.6	107	1.7	90	1.3	41	1.4	15	1.5	12	0.6	7	0.4
