# Supplementary material for: Incidence of viral hepatitis in Brazil from 2009 to 2018: an epidemiological study of confirmed cases of viral hepatitis
Source: Rev Soc Bras Med Trop. 2020 Dec 21;54:e00892020. doi: 10.1590/0037-8682-0089-2020 (PMC7747823; doi:10.1590/0037-8682-0089-2020)
Supplement: Supplementary file 2 [file 1678-9849-rsbmt-54-e00892020-suppl2.pdf]

Webappendix Table 2. Descriptive profile of the population with HBV.

	2009 n=12817		2010 n=12303		2011 n=13698		2012 n=13620		2013 n=16666		2014 n=16863		2015 n=14887		2016 n=14475		2017 n=13379		2018 n=10361	
	**n**	**%**	**n**	**%**	**n**	**%**	**n**	**%**	**n**	**%**	**n**	**%**	**n**	**%**	**n**	**%**	**n**	**%**	**n**	**%**
**Region**																				
North	1762	13.7	1650	13.4	1866	13.6	1794	13.2	3076	18.5	3189	18.9	2141	14.4	2255	1.6	2224	16.6	1774	17.1
Northeast	1232	9.6	1176	9.6	1313	9.6	1370	10.1	1816	10.9	1805	10.7	1574	10.6	1575	10.9	1682	12.6	1391	13.4
Southeast	4632	36.1	4356	35.4	5017	36.6	4911	36.1	5213	31.3	4815	28.6	4791	32.2	4577	31.6	4289	32.1	2930	28.3
South	3789	29.6	3983	32.4	4314	31.5	4392	32.2	5092	30.6	5602	33.2	5102	34.3	4601	31.8	4045	30.2	3518	34.0
Midwest	1402	10.9	1138	9.2	1188	8.7	1153	8.5	1469	8.8	1452	8.6	1279	8.6	1467	10.1	1139	8.5	748	7.2
**Sex**																				
man	6750	52.7	6481	52.7	7234	52.8	7282	53.5	8981	53.9	9086	53.9	8048	54.1	8144	56.3	7454	55.7	5791	55.9
woman	6065	47.3	5820	47.3	6461	47.2	6334	46.5	7682	46.1	7776	46.1	6838	45.9	6330	43.7	5924	44.3	4566	44.1
unknown	2	0.0	2	0.0	3	0.0	4	0.0	3	0.0	1	0.0	1	0.0	1	0.0	1	0.0	4	0.0
**Age group (years)**																				
<10 anos	145	1.1	102	0.8	126	0.9	130	1.0	150	0.9	149	0.9	108	0.7	92	0.6	70	0.5	84	0.8
10-19 anos	821	6.4	690	5.6	697	5.1	616	4.5	681	4.1	601	3.6	439	2.9	339	2.3	315	2.4	155	1.5
20-39 anos	6436	50.2	6033	49.0	6377	46.6	6233	45.8	7700	46.2	7419	44.0	6501	43.7	6129	42.3	5589	41.8	4208	40.6
40 ou mais	5409	42.2	5471	44.5	6493	47.4	6639	48.7	8132	48.8	8690	51.5	7839	52.7	7915	54.7	7405	55.3	5914	57.1
unknown	6	0.0	7	0.1	4	0.0	2	0.0	3	0.0	3	0.0	-	-	-	-	-	-	-	-
**Race**																				
white	6333	49.4	6202	50.4	6668	48.7	6598	48.4	7423	44.5	7609	45.1	6888	46.3	6247	43.2	5534	41.4	4280	41.3
black	896	7.0	897	7.3	905	6.6	967	7.1	1236	7.4	1256	7.4	1275	8.6	1260	8.7	1220	9.1	990	9.6
yellow	303	2.4	180	1.5	153	1.1	217	1.6	230	1.4	230	1.4	229	1.5	174	1.2	188	1.4	126	1.2
mulato	3739	29.2	3667	29.8	4085	29.8	4259	31.3	5859	35.2	6066	36.0	5036	33.8	5189	35.8	5209	38.9	3965	38.3
indigenous	115	0.9	67	0.5	79	0.6	77	0.6	297	1.8	213	1.3	125	0.8	117	0.8	103	0.8	64	0.6
unknown	1431	11.2	1290	10.5	1808	13.2	1502	11.0	1621	9.7	1489	8.8	1334	9.0	1488	10.3	1125	8.4	936	9.0
**Education (years)**																				
illiterate	221	1.7	202	1.6	224	1.6	205	1.5	324	1.9	352	2.1	254	1.7	242	1.7	304	2.3	195	1.9

1 to 4	1919	15.0	1848	15.0	1919	14.0	2001	14.7	2508	15.0	2577	15.3	2065	13.9	2009	13.9	1799	13.4	1410	13.6
5 to 8	3080	24.0	2798	22.7	2921	21.3	2964	21.8	3546	21.3	3521	20.9	3267	21.9	2992	20.7	2716	20.3	2136	20.6
9 to 11	3044	23.7	3031	24.6	3251	23.7	3370	24.7	3993	24.0	4052	24.0	3856	25.9	3622	25.0	3497	26.1	2669	25.8
≥12	917	7.2	928	7.5	1008	7.4	1103	8.1	1312	7.9	1291	7.7	1276	8.6	1216	8.4	1118	8.4	794	7.7
unknown	3636	28.4	3496	28.4	4375	31.9	3977	29.2	4983	29.9	5070	30.1	4169	28.0	4394	30.4	3945	29.5	3157	30.5
**Source of infection**																				
sexual	3192	24.9	3035	24.7	3463	25.3	3390	24.9	4403	26.4	4192	24.9	3569	24.0	3471	24.0	3092	23.1	2354	22.7
transfusion	281	2.2	263	2.1	268	2.0	258	1.9	287	1.7	288	1.7	258	1.7	233	1.6	208	1.6	133	1.3
injection drug use	211	1.6	194	1.6	203	1.5	225	1.7	247	1.5	282	1.7	226	1.5	216	1.5	179	1.3	159	1.5
vertical transmission	307	2.4	345	2.8	366	2.7	451	3.3	566	3.4	569	3.4	473	3.2	407	2.8	332	2.5	241	2.3
work accident	46	0.4	37	0.3	46	0.3	45	0.3	47	0.3	57	0.3	49	0.3	33	0.2	50	0.4	26	0.3
hemodialysis	47	0.4	29	0.2	22	0.2	24	0.2	27	0.2	34	0.2	19	0.1	27	0.2	19	0.1	15	0.1
home	586	4.6	467	3.8	548	4.0	520	3.8	601	3.6	572	3.4	503	3.4	453	3.1	442	3.3	340	3.3
surgical treatment	216	1.7	164	1.3	189	1.4	208	1.5	205	1.2	197	1.2	232	1.6	191	1.3	156	1.2	115	1.1
dental treatment	440	3.4	398	3.2	395	2.9	349	2.6	385	2.3	411	2.4	369	2.5	350	2.4	298	2.2	185	1.8
person to person	161	1.3	194	1.6	228	1.7	308	2.3	326	2.0	503	3.0	371	2.5	383	2.6	367	2.7	306	3.0
oral/fecal	33	0.3	28	0.2	24	0.2	30	0.2	46	0.3	45	0.3	27	0.2	20	0.1	24	0.2	27	0.3
others	370	2.9	347	2.8	375	2.7	388	2.8	493	3.0	494	2.9	477	3.2	436	3.0	514	3.8	415	4.0
unknown	6927	54.0	6802	55.3	7571	55.3	7424	54.5	9033	54.2	9219	54.7	8314	55.8	8255	57.0	7698	57.5	6045	58.3
**Federative unit**																				
Rondônia	411	3.2	423	3.4	475	3.5	500	3.7	759	4.6	699	4.1	663	4.5	621	4.3	549	4.1	407	3.9
Acre	613	4.8	416	3.4	548	4.0	498	3.7	781	4.7	867	5.1	379	2.5	411	2.8	398	3.0	338	3.3
Amazonas	367	2.9	428	3.5	461	3.4	349	2.6	918	5.5	974	5.8	501	3.4	593	4.1	647	4.8	521	5.0
Roraima	96	0.7	83	0.7	97	0.7	98	0.7	77	0.5	113	0.7	92	0.6	129	0.9	87	0.7	112	1.1
Pará	145	1.1	164	1.3	151	1.1	233	1.7	358	2.1	361	2.1	361	2.4	378	2.6	392	2.9	245	2.4
Amapá	17	0.1	27	0.2	17	0.1	22	0.2	20	0.1	28	0.2	33	0.2	54	0.4	49	0.4	38	0.4
Tocantins	113	0.9	109	0.9	117	0.9	94	0.7	163	1.0	147	0.9	112	0.8	69	0.5	102	0.8	113	1.1
Maranhão	207	1.6	200	1.6	245	1.8	186	1.4	201	1.2	208	1.2	197	1.3	195	1.3	176	1.3	184	1.8
Piuaí	21	0.2	25	0.2	24	0.2	51	0.4	72	0.4	72	0.4	43	0.3	47	0.3	60	0.4	44	0.4
Ceará	186	1.5	131	1.1	108	0.8	165	1.2	138	0.8	157	0.9	166	1.1	155	1.1	162	1.2	120	1.2
Rio Grande do Norte	20	0.2	34	0.3	48	0.4	57	0.4	81	0.5	67	0.4	47	0.3	53	0.4	57	0.4	35	0.3
Paraíba	95	0.7	114	0.9	137	1.0	136	1.0	197	1.2	147	0.9	54	0.4	64	0.4	99	0.7	75	0.7
Pernambuco	103	0.8	141	1.1	139	1.0	143	1.0	358	2.1	385	2.3	170	1.1	227	1.6	239	1.8	194	1.9
Alagoas	149	1.2	98	0.8	93	0.7	83	0.6	80	0.5	117	0.7	99	0.7	103	0.7	158	1.2	94	0.9
Sergipe	103	0.8	94	0.8	113	0.8	105	0.8	112	0.7	113	0.7	113	0.8	106	0.7	124	0.9	117	1.1
Bahia	348	2.7	339	2.8	406	3.0	444	3.3	577	3.5	539	3.2	685	4.6	625	4.3	607	4.5	528	5.1
Minas Gerais	750	5.9	695	5.6	733	5.4	611	4.5	754	4.5	936	5.6	926	6.2	824	5.7	772	5.8	615	5.9
Espírito Santo	352	2.7	337	2.7	386	2.8	534	3.9	613	3.7	479	2.8	388	2.6	374	2.6	358	2.7	219	2.1
Rio de Janeiro	688	5.4	633	5.1	886	6.5	717	5.3	736	4.4	586	3.5	597	4.0	598	4.1	526	3.9	271	2.6
São Paulo	2842	22.2	2691	21.9	3012	22.0	3049	22.4	3110	18.7	2814	16.7	2880	19.3	2781	19.2	2633	19.7	1825	17.6

Paraná	1444	11.3	1592	12.9	1755	12.8	1652	12.1	2016	12.1	2039	12.1	1849	12.4	1789	12.4	1670	12.5	1455	14.0
Santa Catarina	1262	9.8	1261	10.2	1415	10.3	1489	10.9	1535	9.2	1728	10.2	1527	10.3	1302	9.0	1051	7.9	882	8.5
Rio Grande do Sul	1083	8.4	1130	9.2	1144	8.4	1251	9.2	1541	9.2	1835	10.9	1726	11.6	1510	10.4	1324	9.9	1181	11.4
Mato Grosso do Sul	275	2.1	186	1.5	165	1.2	143	1.0	195	1.2	162	1.0	123	0.8	111	0.8	133	1.0	108	1.0
Mato Grosso	551	4.3	493	4.0	577	4.2	581	4.3	678	4.1	678	4.0	577	3.9	569	3.9	488	3.6	265	2.6
Goiás	398	3.1	317	2.6	299	2.2	294	2.2	394	2.4	411	2.4	396	2.7	462	3.2	417	3.1	285	2.8
Distrito Federal	178	1.4	142	1.2	147	1.1	135	1.0	202	1.2	201	1.2	183	1.2	325	2.2	101	0.8	90	0.9
