# Supplementary material for: Incidence of viral hepatitis in Brazil from 2009 to 2018: an epidemiological study of confirmed cases of viral hepatitis
Source: Rev Soc Bras Med Trop. 2020 Dec 21;54:e00892020. doi: 10.1590/0037-8682-0089-2020 (PMC7747823; doi:10.1590/0037-8682-0089-2020)
Supplement: Supplementary file 3 [file 1678-9849-rsbmt-54-e00892020-suppl3.pdf]

Webappendix Table 3. Descriptive profile of the population with HCV.

	2009 n=15712		2010 n=15535		2011 n=16940		2012 n=17741		2013 n=19063		2014 n=18225		2015 n=20061		2016 n=19684		2017 n=17253		2018 n=13897	
	n	%	n	%	n	%	n	%	n	%	n	%	n	%	n	%	n	%	n	%
**Region**																				
North	651	4.1	623	4.0	674	4.0	642	3.6	1286	6.7	1038	5.7	931	4.6	1013	5.1	1072	6.2	647	4.7
Northeast	1216	7.7	1112	7.2	1318	7.8	1477	8.3	1771	9.3	1560	8.6	1493	7.4	1549	7.9	1434	8.3	1352	9.7
Southeast	8663	55.1	8424	54.2	9169	54.1	9451	53.3	9177	48.1	8383	46.0	9536	47.5	9485	48.2	7873	45.6	5782	41.6
South	4403	28.0	4782	30.8	5038	29.7	5472	30.8	5955	31.2	6429	35.3	7189	35.8	6565	33.4	6060	35.1	5507	39.6
Midwest	779	5.0	594	3.8	741	4.4	699	3.9	874	4.6	815	4.5	912	4.5	1072	5.4	814	4.7	609	4.4
**Sex**																				
man	9047	57.6	8743	56.3	9376	55.3	9660	54.5	10875	57.0	10422	57.2	11493	57.3	11207	56.9	10046	58.2	7942	57.1
woman	6661	42.4	6786	43.7	7557	44.6	8074	45.5	8165	42.8	7803	42.8	8558	42.7	8473	43.0	7202	41.7	5953	42.8
unknown	4	0.0	6	0.0	7	0.0	7	0.0	23	0.1	-	-	10	0.0	4	0.0	5	0.0	2	0.0
**Age group (years)**																				
<10 anos	112	0.7	96	0.6	130	0.8	120	0.7	125	0.7	118	0.6	115	0.6	111	0.6	105	0.6	86	0.6
10-19 anos	188	1.2	169	1.1	180	1.1	176	1.0	191	1.0	130	0.7	169	0.8	152	0.8	166	1.0	132	0.9
20-39 anos	3940	25.1	3625	23.3	3848	22.7	3772	21.3	3730	19.6	3549	19.5	3484	17.4	3314	16.8	2992	17.3	2353	16.9
40 ou mais	11459	72.9	11635	74.9	12774	75.4	13671	77.1	15013	78.8	14426	79.2	16292	81.2	16106	81.8	13989	81.1	11325	81.5
unknown	13	0.1	10	0.1	8	0.0	2	0.0	4	0.0	2	0.0	1	0.0	1	0.0	1	0.0	1	0.0
**Race**																				
white	9051	57.6	8677	55.9	9235	54.5	9751	55.0	10020	52.6	9690	53.2	10715	53.4	10181	51.7	8995	52.1	7171	51.6
black	1098	7.0	1164	7.5	1212	7.2	1277	7.2	1498	7.9	1362	7.5	1653	8.2	1632	8.3	1468	8.5	1233	8.9
yellow	100	0.6	104	0.7	110	0.6	149	0.8	123	0.6	101	0.6	129	0.6	134	0.7	124	0.7	123	0.9
mulato	3413	21.7	3353	21.6	3664	21.6	3873	21.8	5300	27.8	4823	26.5	5080	25.3	5343	27.1	5014	29.1	3609	26.0
indigenous	31	0.2	32	0.2	22	0.1	38	0.2	50	0.3	47	0.3	53	0.3	39	0.2	52	0.3	38	0.3
unknown	2019	12.9	2205	14.2	2697	15.9	2653	15.0	2072	10.9	2202	12.1	2431	12.1	2355	12.0	1600	9.3	1723	12.4
**Education (years)**																				
illiterate	207	1.3	173	1.1	214	1.3	246	1.4	285	1.5	262	1.4	238	1.2	284	1.4	308	1.8	229	1.6
1 to 4	2241	14.3	2110	13.6	2153	12.7	2257	12.7	2581	13.5	2502	13.7	2673	13.3	2671	13.6	2336	13.5	1706	12.3
5 to 8	4065	25.9	3788	24.4	3885	22.9	4160	23.4	4376	23.0	4166	22.9	4649	23.2	4333	22.0	3902	22.6	2917	21.0
9 to 11	3240	20.6	3107	20.0	3427	20.2	3687	20.8	4222	22.1	3957	21.7	4305	21.5	4195	21.3	3774	21.9	2917	21.0

≥12	1292	8.2	1240	8.0	1315	7.8	1376	7.8	1439	7.5	1272	7.0	1621	8.1	1573	8.0	1377	8.0	1138	8.2
unknown	4667	29.7	5117	32.9	5946	35.1	6015	33.9	6160	32.3	6066	33.3	6575	32.8	6628	33.7	5556	32.2	4990	35.9
**Source of infection**																				
sexual	1359	8.6	1430	9.2	1457	8.6	1441	8.1	1793	9.4	1699	9.3	1963	9.8	2075	10.5	1867	10.8	1404	10.1
transfusion	1783	11.3	1786	11.5	1859	11.0	1815	10.2	1850	9.7	1606	8.8	1679	8.4	1623	8.2	1363	7.9	974	7.0
injection drug use	2117	13.5	1906	12.3	2013	11.9	2077	11.7	2313	12.1	2219	12.2	2242	11.2	1964	10.0	1656	9.6	1272	9.2
vertical transmission	46	0.3	33	0.2	36	0.2	57	0.3	52	0.3	62	0.3	47	0.2	44	0.2	36	0.2	38	0.3
work accident	94	0.6	81	0.5	73	0.4	85	0.5	73	0.4	71	0.4	65	0.3	65	0.3	66	0.4	46	0.3
hemodialysis	92	0.6	84	0.5	135	0.8	101	0.6	144	0.8	107	0.6	111	0.6	140	0.7	84	0.5	95	0.7
home	67	0.4	61	0.4	84	0.5	78	0.4	82	0.4	82	0.4	99	0.5	87	0.4	81	0.5	111	0.8
surgical treatment	636	4.0	657	4.2	680	4.0	837	4.7	742	3.9	697	3.8	725	3.6	666	3.4	533	3.1	343	2.5
dental treatment	498	3.2	480	3.1	539	3.2	586	3.3	497	2.6	376	2.1	480	2.4	413	2.1	351	2.0	280	2.0
person to person	73	0.5	104	0.7	134	0.8	171	1.0	191	1.0	200	1.1	210	1.0	232	1.2	251	1.5	200	1.4
oral/fecal	7	0.0	7	0.0	10	0.1	8	0.0	12	0.1	11	0.1	9	0.0	12	0.1	12	0.1	12	0.1
others	617	3.9	594	3.8	652	3.8	690	3.9	783	4.1	713	3.9	769	3.8	780	4.0	719	4.2	603	4.3
unknown	8323	53.0	8312	53.5	9268	54.7	9795	55.2	10531	55.2	10382	57.0	11662	58.1	11583	58.8	10234	59.3	8519	61.3
**Federative unit**																				
Rondônia	65	0.4	90	0.6	112	0.7	137	0.8	170	0.9	159	0.9	176	0.9	176	0.9	168	1.0	122	0.9
Acre	193	1.2	156	1.0	141	0.8	112	0.6	509	2.7	295	1.6	182	0.9	126	0.6	169	1.0	95	0.7
Amazonas	180	1.1	178	1.1	256	1.5	194	1.1	376	2.0	369	2.0	243	1.2	316	1.6	312	1.8	256	1.8
Roraima	28	0.2	10	0.1	20	0.1	5	0.0	20	0.1	16	0.1	15	0.1	17	0.1	15	0.1	19	0.1
Pará	100	0.6	135	0.9	74	0.4	135	0.8	151	0.8	128	0.7	232	1.2	307	1.6	311	1.8	109	0.8
Amapá	36	0.2	28	0.2	27	0.2	23	0.1	27	0.1	20	0.1	33	0.2	28	0.1	48	0.3	22	0.2
Tocantins	49	0.3	26	0.2	44	0.3	36	0.2	33	0.2	51	0.3	50	0.2	43	0.2	49	0.3	24	0.2
Maranhão	193	1.2	143	0.9	223	1.3	164	0.9	129	0.7	117	0.6	133	0.7	98	0.5	88	0.5	119	0.9
Piuaí	17	0.1	32	0.2	40	0.2	50	0.3	64	0.3	64	0.4	64	0.3	47	0.2	43	0.2	22	0.2
Ceará	166	1.1	168	1.1	147	0.9	182	1.0	184	1.0	192	1.1	233	1.2	211	1.1	154	0.9	111	0.8
Rio Grande do Norte	81	0.5	80	0.5	82	0.5	81	0.5	106	0.6	84	0.5	77	0.4	81	0.4	83	0.5	53	0.4
Paraíba	60	0.4	105	0.7	54	0.3	61	0.3	112	0.6	104	0.6	58	0.3	80	0.4	94	0.5	79	0.6
Pernambuco	165	1.1	139	0.9	244	1.4	266	1.5	454	2.4	376	2.1	178	0.9	204	1.0	223	1.3	152	1.1
Alagoas	55	0.4	45	0.3	60	0.4	49	0.3	45	0.2	59	0.3	85	0.4	70	0.4	108	0.6	53	0.4
Sergipe	56	0.4	47	0.3	66	0.4	69	0.4	75	0.4	57	0.3	60	0.3	74	0.4	64	0.4	68	0.5
Bahia	423	2.7	353	2.3	402	2.4	555	3.1	602	3.2	507	2.8	605	3.0	684	3.5	577	3.3	695	5.0
Minas Gerais	830	5.3	923	5.9	1014	6.0	775	4.4	927	4.9	1187	6.5	1301	6.5	1183	6.0	1001	5.8	880	6.3
Espírito Santo	134	0.9	104	0.7	113	0.7	165	0.9	260	1.4	223	1.2	210	1.0	228	1.2	229	1.3	126	0.9
Rio de Janeiro	1454	9.3	1633	10.5	1730	10.2	2048	11.5	1815	9.5	1407	7.7	1674	8.3	1599	8.1	1225	7.1	612	4.4
São Paulo	6245	39.7	5764	37.1	6312	37.3	6463	36.4	6175	32.4	5566	30.5	6351	31.7	6475	32.9	5418	31.4	4164	30.0
Paraná	846	5.4	955	6.1	1073	6.3	927	5.2	1092	5.7	1175	6.4	1409	7.0	1263	6.4	1196	6.9	943	6.8
Santa Catarina	848	5.4	826	5.3	1026	6.1	993	5.6	1085	5.7	1109	6.1	1125	5.6	1024	5.2	952	5.5	854	6.1

Rio Grande do Sul	2709	17.2	3001	19.3	2939	17.3	3552	20.0	3778	19.8	4145	22.7	4655	23.2	4278	21.7	3912	22.7	3710	26.7
Mato Grosso do Sul	259	1.6	184	1.2	214	1.3	191	1.1	227	1.2	218	1.2	115	0.6	141	0.7	179	1.0	147	1.1
Mato Grosso	120	0.8	117	0.8	190	1.1	189	1.1	250	1.3	218	1.2	192	1.0	162	0.8	205	1.2	144	1.0
Goiás	127	0.8	120	0.8	120	0.7	139	0.8	208	1.1	235	1.3	362	1.8	413	2.1	334	1.9	236	1.7
Distrito Federal	273	1.7	173	1.1	217	1.3	180	1.0	189	1.0	144	0.8	243	1.2	356	1.8	96	0.6	82	0.6
